# The effect of post-traumatic growth on recovery in liver transplant recipients

**DOI:** 10.3389/fpsyt.2023.1150385

**Published:** 2023-05-18

**Authors:** Pinar Harmanci, Semra Bulbuloglu

**Affiliations:** ^1^Division of Psychiatry Nursing, Nursing Department, Faculty of Health Sciences, Kahramanmaras Istiklal University, Kahramanmaras, Turkey; ^2^Division of Surgical Nursing, Nursing Department, Faculty of Health Sciences, Istanbul Aydin University, Istanbul, Turkey

**Keywords:** liver transplant recipients, post-traumatic growth, liver transplantation, recovery, recipient

## Abstract

**Aim:**

In our study, we examined the effect of post-traumatic growth on recovery in liver transplant recipients in the post-transplant period.

**Method:**

This research was performed as a descriptive and cross-sectional study with the participation of 218 patients who had liver transplantation at the liver transplant institute of a research and training hospital. The personal information form, the Post-Traumatic Growth Inventory, and the Recovery Assessment Scale were used in the data collection process. The Statistical Package for Social Science 25.0 was utilized in the data analysis process.

**Findings:**

In the research, of all participant liver transplant recipients, 67.8% were aged 45–64 years, 34.4% had incomes below expenses, and 91.7% had living donor liver transplantation. Besides, it was found that participants who had living donor liver transplantation obtained higher mean scores from both the Post-Traumatic Growth Inventory and the Recovery Assessment Scale than participants who had cadaveric donor liver transplantation, and likewise, participants who had past surgery experiences obtained higher mean scores from both the Post-Traumatic Growth Inventory and the Recovery Assessment Scale than participants who had no past surgery experience (*p* < 0.05). Moreover, there was a statistically significant positive linear relationship between participant liver transplant recipients’ Post-Traumatic Growth Inventory and Recovery Assessment Scale scores.

**Conclusion:**

Post-traumatic growth supports recovery. Also, social support and a good economic situation are other parameters that promote recovery. In the two-year process during which the treatment is intensively applied to liver transplant recipients following the transplantation surgery, it is important to enable patients to find more meaning in life and to find solutions that facilitate recovery.

## Introduction

The health risk caused by physical injuries and psychological traumas can lead to psychiatric problems such as post-traumatic stress disorder, depression, and anxiety (1–4). On the other hand, being exposed to a serious trauma can result in a spectacular transformation in an individual’s life. This spectacular transformation can alter the individual’s perception of life, and by strengthening the coping mechanisms, it can enhance the individual’s psychological resilience, and hence, post-traumatic growth takes place (5). The increase in cognitive functions of an individual who is exposed to a high-level crisis and the rise in the individual’s mental abilities through inner stimulation can be defined as post-traumatic growth (1). In previous studies, it was noted that individuals experienced post-traumatic growth after recovering from diseases that had life-threatening risks (6–9). When individuals who are exposed to serious traumas focus on good deeds by virtue of receiving social support and having a positive mental state, their hopes for living increase, and they can have a larger number of mental achievements. On the other hand, it was found that there was a relationship between the negative mental state and worsening health (1, 2).

One of the patient groups that are most in need of the hope for living and psychological well-being is the individuals that had liver transplantation. Liver transplantation which is used as a life-saving surgical treatment option in end-stage liver diseases is applied frequently across the world. As per 2017 data, the total number of liver transplants in the United States was reported as 8.082 (10). The number of transplants that was 908 in 2011 in Turkey reached 1.776 in 2019 (11). Liver transplantation is a quite complex process that affects the patient and the patient’s family psychosocially. Following the liver transplantation, fear of death and helplessness lead to negative emotions and thoughts in patients. The thinking that the patient will lose physical strength, health, sex life, family order, and ability to work and also the patient’s autonomy will be restricted is among the reasons why patients feel high-level stress in the pre-transplant period (12). In a previous study, it was identified that, as liver transplant recipients’ perceived social support levels increased, their psychological resilience levels also increased. In the same study, it was discerned that liver transplant recipients had medium-level psychological resilience and low-level social support perception (13).

The complications observed frequently due to immunosuppressive therapy following the transplantation surgery can be listed as neuropsychiatric disorders, renal problems, endocrine disorders, blood and heart problems, gastrointestinal problems, edema, malignancy, infections, neurotoxicity, and nephrotoxicity. Also, as per the relevant literature, diabetes, hypertension, coronary artery disease, goiter, and chronic kidney disease are quite prevalent following liver transplantation (13–15). A hard and challenging care process awaits liver transplant recipients in the post-transplant period. Liver patients awaiting the medically suitable organ donor in the pre-transplant period experience a stressful phase with health risks. In the post-transplant period, the new care and treatment protocol, the decrease in functionality, complications, social isolation, and economic problems can be the beginning of new traumas, on the other hand, post-traumatic growth can also be achieved in the same period. In our study, we examined the effect of post-traumatic growth on recovery in liver transplant recipients in the post-transplant period.

## Materials and method

Our research was conducted as a descriptive and cross-sectional study to explore the effect of post-traumatic growth on recovery in patients who had liver transplantation.

### Design and participants

Our study was performed at the liver transplant institute of a research and training hospital located in eastern Turkey. The purposive sampling method was used in the sample selection process. The research sample was comprised of 218 liver transplant recipients. Data was collected by the researchers between 03 November 2021 and 1 June 2022. The inclusion and exclusion criteria designated for participation in this study are as below:

### Inclusion and exclusion criteria

Inclusion criteria included (i) Having had liver transplantation, (ii) Being aged 18 years or above, (iii) Having no speaking, communication, or language barrier, (iv) Volunteering to participate in the study. The exact opposite of the inclusion criteria our exclusion criteria.

### Data collection method and tools

The Personal Information Form, the Post-Traumatic Growth Inventory, and the Recovery Assessment Scale were used in the data collection process. Information about data collection tools is presented below:

### Personal information form

The personal information form is a form that has questions about the personal characteristics of participant patients who had liver transplantation, such as age, gender, marital status, and occupation. Besides these questions, the form contained questions about liver transplantation surgery.

### Post-traumatic growth inventory

The Post-Traumatic Growth Inventory (PTGI) PTGI that was developed by Tedeschi and Calhoun (1996) is a tool of self-report for positive changes occurring after a traumatic experience (16). Designed as a six-point Likert-type scale, the PTGI has 21 items. Kagan et al. performed the validity and reliability study for the PTGI in Turkish. Minimum and maximum scores to be obtained from the PTGI are, respectively, 0 and 105 points (17). A high total PTGI score refers to a high post-traumatic growth level. The PTGI has three sub-scales, that is, “Changes in Self-Perception,” “Changes in Philosophy of Life,” and “Changes in Relationship.” Internal consistency coefficients were calculated as 0.88, 0.78, and 0.77 successively for the above PTGI sub-scales (17). In the current study, internal consistency coefficients were calculated as 0.83, 0.89, 0.82, and 0.82 consecutively for the PTGI and its above sub-scales.

### Recovery assessment scale

The original version of the Recovery Assessment Scale (RAS) has 41 items while its current version is composed of 24 items. The RAS was first developed in the United Kingdom, and then, in its Japanese adaptation, a five-factor version was created. These factors can be listed as “Personal Confidence and Hope,” “Willingness to Ask for Help,” “Goal and Success Orientation,” “Reliance on Others,” and “Not Dominated by Symptoms.” The RAS is scored on the basis of a five-point Likert scale (1: Strongly disagree, 5: Strongly agree). In the validity and reliability study performed for the RAS in Japan, robust results were obtained (Chronbach *α* = 0.89) (18). Güler performed the validity and reliability study for the RAS in Turkey in 2017 (19). In the study by Güler, Cronbach’s alpha coefficients were calculated successively as 0.84, 0.87, 0.84, 0.74, and 0.89 for the above RAS sub-scales (19). In our study, Cronbach’s alpha coefficient was calculated as 0.91 for the RAS.

### Statistical analysis

The Statistical Package for Social Science (SPSS) 25.0 was utilized in the data analysis process. Before the analysis, by using the Kolmogorov–Smirnov test, it was identified that the research data were normally distributed. Moreover, frequencies, percentages, arithmetic means, and standard deviations were used as the descriptive statistics. The independent samples t-test and one-way analysis of variance (ANOVA) were used for group comparisons, and the *post hoc* analysis was utilized additionally in the identification of statistically significant differences. We used correlation analysis to compare scale scores. We used scatter plot and regression line while creating the figure. At a confidence interval of 95%, results were evaluated with the 5% significance level (*p* < 0.05).

### Ethical aspect of the research

Before the research, the relevant permissions were received from the Ethics Committee of the Faculty of Medicine of Inonu University (Date: 02.11.2021, Number of Sessions: 22, Number of decisions: 2021/2607) and the Office of the Chief Physician of Turgut Ozal Research and Training Hospital in Turkey. An informed consent form designed in compliance with the principles of the Declaration of Helsinki was submitted by each liver patient. It was informed that the study was a descriptive study, that no attempt would be made, and that identity information would not be obtained. Patients (liver transplant recipients) volunteering to participate in the study were included in the research only after they submitted the informed consent form.

### Findings

Table 1 displayed participant liver transplant recipients’ personal characteristics and mean PTGI and RAS scores.

**Table 1:** Liver transplant recipients’ descriptive characteristics and mean PTGI and RAS scores (*n* = 218).

Descriptive characteristics	*n*	%	PTGI	RAS
Age			**Mean ± SD**	**Mean ± SD**
30–44 years (1)	52	23.9	60.42 ± 22.98	76.67 ± 23.41
45–64 years (2)	148	67.8	64.68 ± 20.09	83.20 ± 21.21
65 years or above (3)	18	8.3	63.66 ± 14.73	76.22 ± 19.53
Test and sig.			*F* = 0.955	*F* = 1.328
			*p* = 0.645	*p* = 0.016*
*Post Hoc*				2 > 1,3
*Gender*				
Female	57	26.1	63.89 ± 22.48	79.73 ± 22.44
Male	161	73.9	63.47 ± 19.74	81.65 ± 21.56
Test and sig.			*t* = 1.654	*t* = 0.628
			*p* = 0.111	*p* = 0.979
*Marital status*				
Married	163	74.8	63.02 ± 20.27	80.28 ± 21.61
Single	55	25.2	65.03 ± 21.04	83.40 ± 22.23
Test and sig.			*t* = 0.945	*t* = 1.168
			*p* = 0.645	*p* = 0.225
*Education level*				
Literate (1)	35	16.1	67.22 ± 17.92	82.25 ± 22.54
Primary school (2)	48	22	59.54.19.18	77.45 ± 19.87
High school (3)	70	32.1	62.25 ± 23.09	81.08 ± 21.18
University or higher education (4)	65	29.9	67.88 ± 19.38	84.22 ± 26.03
Test and sig.			*F* = 1.021	*F* = 1.127
			*p* = 0.449	*p* = 0.292
*Post Hoc*				
*Income level*				
Income below expenses (1)	75	34.4	63.36 ± 21.23	68.04 ± 21.62
Income equaling expenses (2)	90	41.3	62.56 ± 20.18	79.92 ± 22.55
Income above expenses (3)	53	24.3	65.62 ± 19.95	88.49 ± 20.58
Test and sig.			*F* = 1.083	*F* = 0.745
			*p* = 0.343	*p* = 0.001^**^
*Post Hoc*				3 > 1,2
*Occupation*				
Civil servant	57	26.1	62.08 ± 20.84	82.43 ± 22.21
Worker	43	19.7	62.27 ± 17.31	83.95 ± 21.22
Self-employed	54	24.8	62.37 ± 19.69	77.11 ± 21.52
Housewife	34	15.6	63.64 ± 23.94	83.47 ± 24
Retired	20	9.2	63.80 ± 21.24	81.70 ± 16.81
Not working	10	4.6	62.10 ± 23.31	72.90 ± 23.75
Test and sig.			*F* = 1.260	*F* = 1.229
			*p* = 0.132	*p* = 0.160
*Past surgery experience*				
Yes	95	43.6	65.15 ± 20.19	83 ± 20.43
No	123	56.3	62.10 ± 17.48	77.40 ± 18.63
Test and sig.			*t* = 1.023	*t* = 1.328
			*p* = 0.024^*^	*p* = 0.018^*^
*Donor type*				
Living donor	200	91.7	63.85 ± 20.08	81.40 ± 21.41
Cadaveric donor	18	8.3	60.61 ± 24.55	77.38 ± 25.65
Test and sig.			*t* = 2.500	*t* = 1.015
			*p* = 0.000^**^	*p* = 0.012^*^
*Transplant time*				
0–6 months (1)	24	11	62.34 ± 14.73	72.51 ± 19.51
6–12 months (2)	16	7.3	59.34 ± 18.34	79.36 ± 17.36
1–2 years (3)	56	25.7	66.14 ± 18.11	82.09 ± 20.24
More than 2 years (4)	122	56	69.81 ± 18.33	85.24 ± 24.03
Test and sig.			*F* = 0.897	*F* = 658
			*p* = 0.014^*^	*p* = 0.030^*^
PostHoc			3,4 > 1,2	3,4 > 1,2
*Means*				
Age (year)		51.67 ± 7.93	Min, max (36, 72)	
Number of days following liver transplantation		299 ± 31.25	Min, max (14, 567)	

Sig, Significance; SD, Standard Deviation. ^*^p < 0.05, ^**^p < 0.01.

*t*; Chi-Squared test, F; One-way analysis of variance (ANOVA).Bold *p* values express statistical significance. For this *p.

In the research, of all participant liver transplant recipients, 67.8% were aged 45–64 years, 73.9% were male, 32.1% were high school graduates, 41.3% had incomes equaling their expenses, 26.1% were civil servant, 56.3% had no past surgery experience, and 91.7% had living donor liver transplantation. The mean number of days spent by liver transplant recipients following transplantation surgery was 299 ± 31.25 days (min 14, max 567). The proportion of recipients who had a liver transplant more than 2 years ago was 56%.

In our study, liver transplant recipients’ mean PTGI and RAS scores were analyzed and compared as per their personal characteristics. In this respect, it was found that liver transplant recipients’ personal characteristics had no statistically significant effect on their PTGI scores. It was discerned that participants who had living donor liver transplantation obtained higher mean PTGI and RAS scores than participants who had cadaveric donor liver transplantation, and likewise, participants who had past surgery experiences obtained higher mean PTGI and RAS scores than participants who had no past surgery experience (*p* < 0.05). Moreover, it was identified that participants who were aged 45–64 years and had incomes above their expenses had higher mean RAS scores than other corresponding groups of participants and this difference between them was statistically significant. Liver recipients with a transplant time of more than 1 year had higher PTGI and RAS scores than others.

Table 2 exhibited mean scores obtained by participant liver transplant recipients from the PTGI, PTGI sub-scales, the RAS, and RAS sub-scales. In this regard, liver transplant recipients’ mean PTGI score was 63.58 ± 20.44 points while mean scores obtained by liver transplant recipients from PTGI sub-scales of “Changes in Self-Perception,” “Changes in Philosophy of Life,” and “Changes in Relationship” were successively 33.15 ± 10.58, 18.39 ± 6.16, and 12.02 ± 6.45 points. Furthermore, liver transplant recipients’ mean RAS score was 81.07 ± 21.75 points while mean scores obtained by liver transplant recipients from RAS sub-scales of “Personal Confidence and Hope,” “Willingness to Ask for Help,” “Goal and Success Orientation,” “Reliance on Others,” and “Not Dominated by Symptoms” were, respectively, 30.84 ± 8.15, 10.27 ± 2.95, 16 ± 4.59, 13.72 ± 3.76, and 10.22 ± 2.91 points.

**Table 2:** Liver transplant recipients’ total and sub-dimensions mean PTGI and RAS scores (*n* = 218).

Scales and sub-scales	Number of items	Items	Score range	Min.-Max.	Mean ± SD
PTGI	21	1–21	0–105	6–102	63.58 ± 20.44
Changes in self-perception	10	5,10,11,12,13,15,16,17,18,19	0–50	0–50	33.15 ± 10.58
Changes in philosophy of Life	6	1,2,3,4,7,14	0–30	1–30	18.39 ± 6.16
Changes in relationship	5	6,8,9,20,21	0–25	0–24	12.02 ± 6.45
RAS	24	1–24	24–120	40–120	81.07 ± 21.75
Personal confidence and Hope	9	7, 8, 9, 10, 11, 12, 13, 14, 21	9–45	15–45	30.84 ± 8.15
Willingness to ask for help	3	18, 19, 20	3–15	4–15	10.27 ± 2.95
Goal and success orientation	5	1, 2, 3, 4, 5	5–25	7–25	16 ± 4.59
Reliance on others	4	6, 22, 23, 24	4–20	6–20	13.72 ± 3.76
Not dominated by symptoms	3	15, 16, 17	3–15	4–15	10.22 ± 2.91

**Figure 1:**
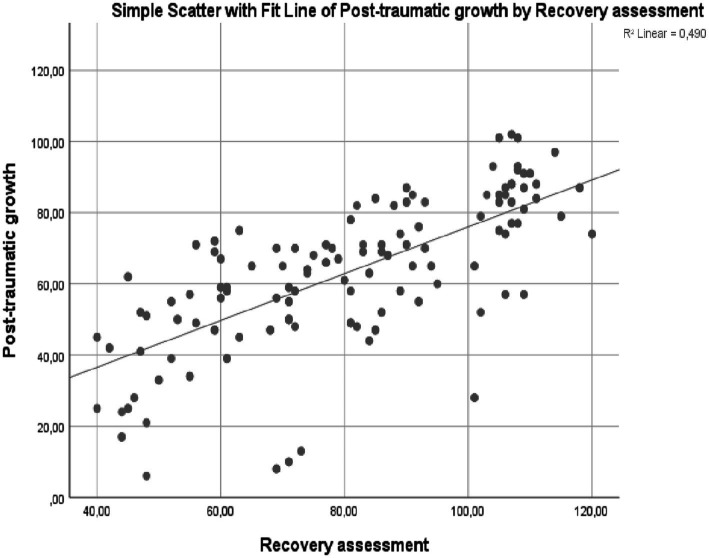
Scatter plot and regression line indicating the relationship between liver transplant recipients’ PTGI and RAS scores (*n* = 218).

Table 3 showed the results of correlation analysis conducted on liver transplant recipients’ PTGI and RAS scores. According to Table 3, there was a positive relationship between participant liver transplant recipients’ PTGI and RAS scores, and this relationship was statistically significant. The sub-dimensions of PTGI and RAS were also in a positive and statistically significant relationship with each other. In Figure 1, the relationship between liver transplant recipients’ PTGI and RAS scores was expressed as a scatter plot and regression line. Accordingly, the relationship between PTGI and RAS is that both increase each other in the same direction.

**Table 3:** Correlation matrix conducted on liver transplant recipients’ PTGI and RAS scores (*n* = 218).

		1	2	3	4	5	6	7	8	9	10
PTGI (1)	Pearson Correlation	1	0.940^**^	0.850^**^	0.813^**^	0.700^**^	0.681^**^	0.696^**^	0.701^**^	0.687^**^	0.627^**^
	Sig. (2-tailed)		0.000	0.000	0.000	0.000	0.000	0.000	0.000	0.000	0.000
	*N*	218	218	218	218	218	218	218	218	218	218
Changes in self-perception (2)	Pearson Correlation	0.940^**^	1	0.727^**^	0.644^**^	0.687^**^	0.668^**^	0.687^**^	0.689^**^	0.672^**^	0.611^**^
	Sig. (2-tailed)	0.000		0.000	0.000	0.000	0.000	0.000	0.000	0.000	0.000
	*N*	218	218	218	218	218	218	218	218	218	218
Changes in philosophy of life (3)	Pearson Correlation	0.850^**^	0.727^**^	1	0.544^**^	0.614^**^	0.608^**^	0.605^**^	0.617^**^	0.592^**^	0.551^**^
	Sig. (2-tailed)	0.000	0.000		0.000	0.000	0.000	0.000	0.000	0.000	0.000
	*N*	218	218	218	218	218	218	218	218	218	218
Changes in relationship (4)	Pearson Correlation	0.813^**^	0.644^**^	0.544^**^	1	0.504^**^	0.481^**^	0.501^**^	0.500^**^	0.508^**^	0.457^**^
	Sig. (2-tailed)	0.000	0.000	0.000		0.000	0.000	0.000	0.000	0.000	0.000
	*N*	218	218	218	218	218	218	218	218	218	218
RAS (5)	Pearson Correlation	0.700^**^	0.687^**^	0.614^**^	0.504^**^	1	0.955^**^	0.978^**^	0.986^**^	0.960^**^	0.961^**^
	Sig. (2-tailed)	0.000	0.000	0.000	0.000		0.000	0.000	0.000	0.000	0.000
	*N*	218	218	218	218	218	218	218	218	218	218
Not dominated by symptoms (6)	Pearson Correlation	0.681^**^	0.668^**^	0.608^**^	0.481^**^	0.955^**^	1	0.917^**^	0.931^**^	0.918^**^	0.896^**^
	Sig. (2-tailed)	0.000	0.000	0.000	0.000	0.000		0.000	0.000	0.000	0.000
	*N*	218	218	218	218	218	218	218	218	218	218
Reliance on others (7)	Pearson Correlation	0.696^**^	0.687^**^	0.605^**^	0.501^**^	0.978^**^	0.917^**^	1	0.951^**^	0.952^**^	0.931^**^
	Sig. (2-tailed)	0.000	0.000	0.000	0.000	0.000	0.000		0.000	0.000	0.000
	*N*	218	218	218	218	218	218	218	218	218	218
Personal confidence and hope (8)	Pearson Correlation	0.701^**^	0.689^**^	0.617^**^	0.500^**^	0.986^**^	0.931^**^	0.951^**^	1	0.930^**^	0.929^**^
	Sig. (2-tailed)	0.000	0.000	0.000	0.000	0.000	0.000	0.000		0.000	0.000
	*N*	218	218	218	218	218	218	218	218	218	218
Willingness to ask for help (9)	Pearson Correlation	0.687^**^	0.672^**^	0.592^**^	0.508^**^	0.960^**^	0.918^**^	0.952^**^	0.930^**^	1	0.890^**^
	Sig. (2-tailed)	0.000	0.000	0.000	0.000	0.000	0.000	0.000	0.000		0.000
	*N*	218	218	218	218	218	218	218	218	218	218
Goal and success orientation (10)	Pearson Correlation	0.627^**^	0.611^**^	0.551^**^	0.457^**^	0.961^**^	0.896^**^	0.931^**^	0.929^**^	0.890^**^	1
	Sig. (2-tailed)	0.000	0.000	0.000	0.000	0.000	0.000	0.000	0.000	0.000	
	*N*	218	218	218	218	218	218	218	218	218	218

** Correlation is significant at the 0.01 level (2-tailed).

## Discussion

In the post-transplant period, the immunosuppressive drug protocol necessitating care and attention, the advanced-level hygiene need due to the suppressed immune system, the pain, the fear of death, drug side effects, the care dependency, and the psychological burden imposed by the patient’s failure to protect his/her autonomy are crucial sources of stress (20). In a previous study, it was emphasized that there was a noticeable deterioration in liver transplant recipients’ daily life activities following liver transplantation (14). Challenges experienced after liver transplantation delay the recovery of liver transplant recipients. In our study, it was discerned that liver transplant recipients had medium-level recovery scores. In a study that was performed with liver transplant recipients who lived for 9 years following liver transplantation, it was found that the good general health state and high quality of life were positively correlated with post-traumatic growth (20). High quality of life is certainly affected by the high economic level. In our study, it was discerned that liver transplant recipients with incomes above expenses had higher levels of recovery.

In the period of a minimum of 2 years following liver transplantation, patients need more economic and psychosocial assistance. Quality of life and comfort levels decrease and more monetary support is needed as patients cannot work and depend most of the time on the caregiver. Adaptation to immunosuppressive drug therapy and other treatment protocols can be, first of all, achieved with material ease and comfort as patients who had liver transplantation need to have better care and more rest and repose abundantly. Few studies in the literature have linked economic deficiencies with poor health outcomes (cardiovascular disease, serious diabetes complications, high cancer prevalence and cancer-based mortality risk) (21–23). It was reported that 36.8% of liver transplant recipients had diabetes and 7.9% of them had cardiovascular problems in the period following liver transplantation (24). Liver transplant recipients often suffer from prolonged immunosuppressive therapy, susceptibility to infection, hyperlipidemia, obesity, hypertension, and diabetes mellitus (14, 15, 25). It is inevitable that liver transplant recipients have difficulties in coping with the use of multiple drugs and post-transplant disease burden, and liver transplant surgery involves extremely stressful and difficult processes during the perioperative period. In our study, it is evident that recipients at the risk in terms of comorbidities, diabetes, cardiovascular diseases, and mortality.

Furthermore, in our study, it was found that participants who had living donor liver transplantation obtained both higher post-traumatic growth scores and higher recovery scores than participants who had cadaveric donor liver transplantation (*p* < 0.05). The increase in the number of liver transplant centers today and positive patient outcomes attained in the period following liver transplantation draw attention to the inadequacy of the number of donors. In our study, it was discerned that 91.7% of liver transplant recipients had living donor liver transplantation. Liver transplant recipients who have living donor liver transplantation generally feel deep gratitude toward the organ donor, and the intimacy with the donor ties recipients more tightly to living by convincing them that they are really loved by the people around them, and also, this intimacy will reinforce recipients’ social support perceptions (26, 27). In previous studies, it was identified that kidney patients who had living donor kidney transplantation had higher levels of post-traumatic growth than those who had cadaveric donor kidney transplantation (28, 29). In our study, liver transplant recipients with living donor had higher post-traumatic growth. This may be due to higher perceived social support.

Lastly, in our study, it was found that there was a statistically significant positive relationship between post-traumatic growth and recovery. In the relevant literature, it was put forward that post-traumatic growth improved general health (20) and recovery (30) further in patients who had liver transplantation. Also, it was reported that post-traumatic growth enhanced the patient’s independence, quality of life, productivity, and satisfaction (31). The results of our study are in support of the results in the relevant literature. The fact that our study was a single-center trial and was performed with the participation of the liver transplant receiving population that was in the two-year period when the most intensive treatment protocols were administered following liver transplantation can be accepted as the limitation of our study.

## Conclusion

In the period following liver transplantation, the recovery is affected by post-traumatic growth. As per the findings of our study, it is discerned that liver transplant recipients who were faced with a variety of problems in the perioperative process acquired a life experience on the occasion of each problem, and these experiences were effective in coping with problems, led to cognitive changes, and reinforced liver transplant recipients psychologically. In our study, it was determined that there is a same-aspect relationship between post-traumatic growth and recovery in liver transplant patients. In our study, the finding that having living donor liver transplantation enhanced both recovery and post-traumatic growth can be related to the motivation created by social support. In order to accelerate the recovery of liver recipients, we recommend that attempts be made to raise awareness about posttraumatic growth and that future studies should be built on this.

## Data availability statement

The raw data supporting the conclusions of this article will be made available by the authors, without undue reservation.

## Ethics statement

The studies involving human participants were reviewed and approved by No: 2021/2607. The patients/participants provided their written informed consent to participate in this study.

## Author contributions

SB and PH study conception and design, data collection, and critical revision of the manuscript. SB data analysis and interpretation. PH drafting of the manuscript. All authors contributed to the article and approved the submitted version.

## Conflict of interest

The authors declare that the research was conducted in the absence of any commercial or financial relationships that could be construed as a potential conflict of interest.

## Publisher’s note

All claims expressed in this article are solely those of the authors and do not necessarily represent those of their affiliated organizations, or those of the publisher, the editors and the reviewers. Any product that may be evaluated in this article, or claim that may be made by its manufacturer, is not guaranteed or endorsed by the publisher.
